# Prehospital blood transfusion: 5-year experience of an Australian helicopter emergency medical service

**DOI:** 10.1186/cc12233

**Published:** 2013-03-19

**Authors:** PB Sherren, B Burns

**Affiliations:** 1 Greater Sydney Area HEMS, Sydney, Australia

## Introduction

There is an emerging body of evidence suggesting that early packed red blood cell transfusion accompanied by fresh frozen plasma, while limiting crystalloids, confers a survival benefit in major trauma [1]. Prehospital blood transfusion has been infrequently described, and concerns over expense, transfusion reactions, risk of disease transmission, short shelf half-life and difficult storage have limited the interest of prehospital providers.

## Methods

All Greater Sydney Area HEMS (GSA-HEMS) prehospital missions involving a blood transfusion over a 66-month period were identified and reviewed. The prospectively completed GSA-HEMS electronic database was utilised to identify patients and extract data.

## Results

We identified 158 missions involving a prehospital blood transfusion, of which 147 patient datasets were complete. The majority of patients had a blunt mechanism of injury (93.9%) and were male (69.3%) with a median (IQR) age of 34.5 (22 to 52) years (Table 1). The majority of patients were haemodynamically unstable, with a median (IQR) heart rate and systolic blood pressure of 115 (90 to 130) and 80 (65 to 105) mmHg, respectively. Twenty-two patients (15.0%) were pronounced life extinct on the scene. A total of 382 units of packed red blood cells were transfused, with a median of 3 units (range 1 to 6). No early transfusion reactions were noted. A variety of prehospital interventions accompanied the transfusions, ranging from rapid sequence intubation through to thoracotomies (Table 2).

**Table 1:** Demographics of patients receiving a prehospital blood transfusion

	Total (*n *= 147)
Male (%)	102 (69.3)
Age (years), median (IQR)	34.5 (22 to 52)
Mechanisms of injury (%)	
Motor vehicle collision	87 (59.1)
Motor bike collision	20 (13.6)
Pedestrian versus car	9 (6.1)
Gunshot wound/stabbing	9 (6.1)
Fall from a height	5 (3.4)
Recreational	6 (4.1)
Other	11 (7.5)
Number of patients trapped on arrival (%)	45 (30.6)
Scene time (minutes), mean (SD)	49.9 (27.8)
Time from tasking to arrival at hospital (minutes), mean (SD)	126.5 (51.3)
Heart rate, median (IQR)	115 (90 to 130)
Systolic blood pressure (mmHg), median (IQR)	80 (65 to 105)
RTS_o_^2^, median (IQR)	5.967 (4.083 to 6.904)
Total number of PRBC units transfused	382
Total number of PRBC units wasted	66
Volume of crystalloid (ml), median (IQR)	500 (0 to 1,500)
Pronounced life extinct on scene	22 (15.0)

**Table 2:** Interventions performed

Intervention	Total (*n *= 147)
Rapid sequence intubation	96 (65.3)
Cold endotracheal intubation	15 (10.2)
Surgical airway	1 (0.7)
Thoracostomy (open or tube)	59 (40.1)
Thoracotomy	3 (2.0)
Pelvic binder or fracture splintage	89 (60.5)
Intraosseous insertion sites	22 (15.0)
Humerus	19
Tibia	10
Femur	1
Tourniquet application	15 (10.2)
Hypertonic saline administration	16 (10.9)
E-FAST performed	27 (18.4)
Negative results	9
Positive results (findings)	18
Abdominal free fluid	14
Pneumothorax	2
Haemothorax	1
Thoracic ultrasound only; all negative	2 (1.3)
Cardiac ultrasound only; no cardiac motion	1 (0.7)

## Conclusion

Despite the controversies over the role of fluids in the prehospital environment, the carriage and use of blood is both feasible and safe in a physician-led helicopter emergency medical service.
